# Spontaneous Recovery of Penetrating Cervical Spinal Cord Injury with Physiotherapeutic Treatment: Case Report and Review of the Literature

**DOI:** 10.1155/2021/3741461

**Published:** 2021-12-26

**Authors:** Yao Christian Hugues Dokponou, Mamoune El Mostarchid, Housni Abderrahmane, Niamien Patrice Koffi, Miloudi Gazzaz, Brahim El Mostarchid

**Affiliations:** Department of Neurosurgery, Mohammed V Military Teaching Hospital, Faculty of Medicine and Pharmacy Rabat, Rabat, Morocco

## Abstract

Stab wounds to the cervical spine are less common than injuries from road accidents, sports injuries, and falls. The presence of vital, vascular, neural, respiratory, and digestive structures in the neck region mean that this kind of spinal injury is generally critical, and its management is a challenge. We report a unique case of a previously healthy 17-year-old adolescent admitted for quadriplegia secondary to a stab wound to the cervical spine at the C4C5 level. There was no surgical indication. The patient underwent physiotherapy. He showed spontaneous neurological improvement two weeks later and was able to sit on his own and to walk about three months of physical rehabilitation.

## 1. Introduction

Road accidents, falls, and gunshots are the leading causes of spinal cord injury. These may result from bone fragments impacting and compressing the spinal cord or from concussive shock waves generated by the impact of striking a vertebral body. Different types of injury have different motor recovery outcomes. The global incidence of spinal cord injury has been estimated to range between 15 and 40 individuals per million. Functional outcome is a primary concern for clinicians and patients and is dependent on neurological status [1–3].

Although relatively uncommon, penetrating neck trauma has the potential for serious morbidity and estimated mortality of up to 6%. Correct assessment and management of patients who have sustained a penetrating neck injury have been a controversial issue in the past [4, 5]. Controversy over the surgical treatment of stab wounds to the spinal cord persists, even regarding cases where neurological deficits are apparent.

We report a case of penetrating injury to the cervical spine. This case was unique in two aspects. First, the mechanism was exceptional, with a stab wound to the neck from which the knife had been removed prior to admission. Second, the patient showed spontaneous neurological improvement with physical therapy rehabilitation. After presenting this case, we discuss relevant comparative cases from the literature.

## 2. Case Presentation

A 17-year-old patient was admitted with quadriplegia secondary to a penetrating wound to the cervical spine. The patient reported an assault a few hours earlier resulting in a knife wound to his neck. According to witnesses, a free pocket knife was the assault weapon with the blade oriented vertically. Upon physical examination, he was found to be unable to walk or hold a sitting position. The knife wound entry point was on the right side of the posterior of his neck. Neurological examination found the patient to be alert, with a Glasgow Coma Scale score of 15, and hemodynamically stable. His blood pressure was 130/80 mm Hg, his heart rate was 80 beats/min^−1^, his respiratory rate was 19 mn^−1^, and his body temperature was 37°C. The osteotendinous reflexes were abolished with indifferent cutaneoplantar reflexes. Grade B of the ASIA impairment scale, sensory but no motor function is preserved below the cervical region at the admission. The patient's medical and family histories were unremarkable.

**Figure 1:**
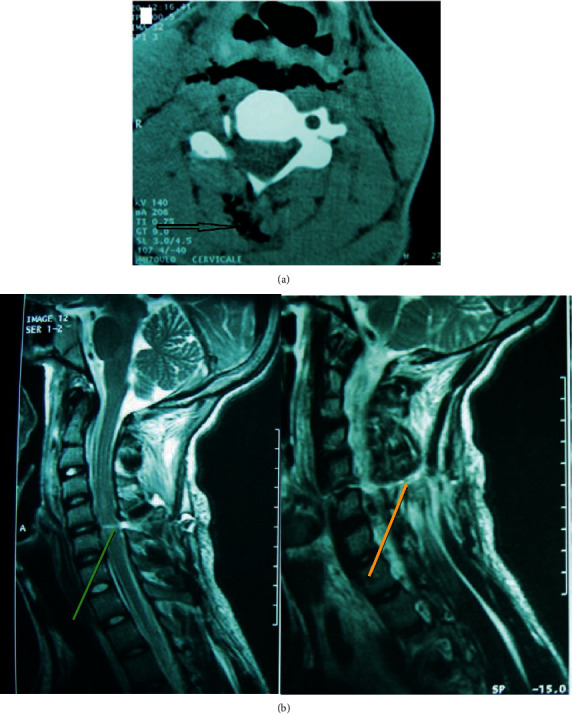
(a) CT scan, axial section at C4C5, showing hypodensity in the posterior left-sided soft tissue representing the knife track with its passage filled with air (black arrow). (b) MRI sagittal T2-weighted hyperintense signal of the spinal cord at C4C5 level sign of medullary contusion (green arrow), with the continuous posterior soft tissue hyperintense representing the knife track (yellow arrow).

A computed tomography (CT) scan at 4 hours after the injury showed the path of the knife. This was filled with air, and there was no apparent injury to the visceral structures of the neck (Figure 1(a)). A magnetic resonance imaging (MRI) scan of the cervical spine at the admission (about 10 hours from the assault) also showed the knife track and revealed spinal cord contusion at the C4C5 level (Figure 1(b)). Routine laboratory tests of the patient's blood sample were normal.

There were no secondary complications, such as meningitis or worsening of neurological deficits. The patient did not undergo surgery but benefited from a series of physical therapy, 30 sessions of 30 minutes each on daily basis aiming at functional recovery. The functional electrical stimulation was not used. The main physiotherapy treatment were muscles stretching, strengthening for elasticity and mobility. This allowed to reinforce the muscles and teach the patient the control and usage of his limbs. He showed spontaneous neurological improvement and regain the usage of his arms and hands as well as the lower limbs. The medical treatment was made of pain relievers (paracetamol + tramadol) and empirical antibiotic (third generation cephalosporins, rocephin 2 g/day for two weeks + metronidazole 500 mg twice a day for 10 days). After two weeks, he was able to sit unaided and walked at three months of physiotherapy.

## 3. Discussion

Our study finds that a patient with neurological deficit secondary to a penetrating spinal injury can be completely recovered with a constant and repeated series of physiotherapy rehabilitation treatment. There was no foreign material to be taking care of, no hemorrhage, no life-threatening sign, and no spinal stability lesion; therefore, no indication for surgery. But taking into account the patient's neurological status, following this spinal trauma, one might be tempted to carry out surgery for exploration and decompression.

We have reviewed other cases of penetrative spinal cord injuries to determine appropriate management and treatment. Our findings are discussed, and the cases identified are given in Table 1.

The initial management of penetrating spinal injury follows the standard principles of trauma care. Once the patient is hemodynamically stabilized and concomitant life-threatening injuries have been addressed, the spinal injury can be treated [16]. Radiological exploration of spinal cord injuries can find anything from severe damage to no harm at all and patients might or might not demonstrate neurologic deficits. This makes the choice of treatment challenging as it is difficult to determine whether or not a patient is a candidate for surgery. There are no clinical findings specific to, and always present in, patients with stab wounds to the spine. Barkana et al. [1] reviewed cases of cervical injury to the neck and found that 22% presented with life-threatening symptoms while others were asymptomatic. The thoracic spine is the most commonly affected area in spinal stab wounds, while such injuries to the cervical spine are rare.

Diagnostic imaging studies used with spinal injuries consist of a standard X-ray and a contrast CT scan. MRI scans are also recommended to define the extent of neural damage. However, with most knife wounds, the latter can only be performed postoperatively because of the metallic nature of the weapon [5]. However, in our case, the knife was not present, so we were able to perform an MRI immediately. The CT scan was not performed until later. MRI can be used to evaluate the damage to the spinal cord, including contusions, hematoma, and compression of extramedullary origin. In stab wounds, intramedullary injuries can include contusion, hematoma, and knife-track damage. A contusion appears as hypointense signals on T1-weighted images and hyperintense signals on T2-weighted images. Oxyhemoglobin reveals acute hemorrhage as isosignals or low-signal intensity on T2-weighted images. Knife-track lesions appear as linear high-signal intensity on T2-weighted images [10]. The MRI in our case showed the knife-track lesion and medullary contusion. Thus, the MRI is the modality of choice for the evaluation of penetrating injuries to the spinal canal in patients with neurological symptoms. Nevertheless, researchers who have reported early use of a CT scan of the cervical spine were able to ascertain that the continuity of the spinal cord had been severely disrupted by penetrative injury [6].

Most authors agree that, depending on the injury, the operative management of penetrating injuries to the cervical spine may focus on the prevention of cerebrospinal fluid fistula or consist of debridement of the devitalized tissues to minimize the chance of infection and decompression of the spinal cord and nerve roots by removing hematomas and bone fragments [17–20]. Burgess et al. [4] suggest that indications for immediate surgical exploration of penetrating neck injuries include airway compromise, massive subcutaneous emphysema, air bubbling through a wound, profuse active bleeding, refractory shock, evolving stroke, and rapidly expanding hematoma. The policy of mandatory surgical exploration for all injuries that breach the platysma is no longer widely practiced. Data from both retrospective and prospective case series support a conservative approach to the management of asymptomatic patients with negative results in physical examinations and imaging investigations. However, there is no evidence-based data in the literature for cases such as ours in which the patient is symptomatic, but physical examination and imaging results are negative. We chose not to operate on our patient, and he began to recover spontaneously from his tetraplegia. This suggests that some patients with neurological deficits may also respond to conservative treatment. Kendall et al. [7] reviewed 218 patients with penetrating neck injuries who underwent surgical exploration. They found that stab wounds had a 10% higher rate of negative exploration than injuries from a projectile. A systematic review and meta-analysis with a large sample is required to gather sufficient data to provide the basis for standardized protocol in the treatment of patients with penetrating injuries to the cervical spine.

## 4. Conclusions

Penetrating stab wounds to the cervical spine are uncommon, and the best protocol for their management is controversial. Nevertheless, we should not rush to operate on patients with penetrating spinal injuries, particularly when there is no obvious indication for surgery. This remains true even when neurological deficits are present.

In the absence of secondary complications, physiotherapy management should be the first-line treatment for penetrative injuries to the spine.

## Figures and Tables

**Table 1:** The management and outcomes of stab wounds to the cervical spinal cord reported in the literature.

First author	Stab wound to cervical spinal cord	Symptomatic	Conservative treatment	Surgical treatment	Outcome (worsened/improved)
Lin et al. [6], 2005	1 case	Yes	—	1 case	Improved
Kendall et al. [7], 1998	218 cases	Yes	—	218 cases	Improved
Barkana et al. [1], 1999	12 cases	Yes	5 cases	7 cases	Improved
Prichayudh et al. [8], 2015	64 cases	40 cases	24 cases	40 cases	Improved
Como et al. [9], 2005	45 cases	Yes	—	45 cases	Eight died
Kamaoui et al. [10], 2007	1 case	Yes	—	1 case	Improved
Dran et al. [11], 2005	2 cases	Yes	—	2 cases	Improved
Harrop et al. [12], 2001	12 cases	Yes	9 cases	3 cases	Three worsened
Wang et al. [13], 2012	1 case	Yes	—	1 case	Improved
Demetriades et al. [14], 1996	89 cases	Yes	—	31 cases	Improved
Ihalainen et al. [3], 2017	168 cases	Yes	142 cases	26 cases	Improved
Demetriades et al. [15], 1997	123 cases	Yes	—	14 cases	Improved
